# Prognostic value of PREVENT, PCE, and social determinants of health for cardiovascular mortality in the Healthy Aging in Neighborhoods of Diversity across the Life Span (HANDLS) study

**DOI:** 10.1186/s12889-026-27358-5

**Published:** 2026-04-29

**Authors:** Carolina G. Downie, Jungnam Joo, Maryam Hashemian, Fang Zhu, Sheila Hernandez-Rubio, Michele K. Evans, Alan B. Zonderman, Véronique L. Roger

**Affiliations:** 1 Heart Disease Phenomics Laboratory, Epidemiology and Community Health Branch, National Heart, Lung, and Blood Institute, National Institutes of Health, NIHBC 10 - Clinical Center BG, 10 Center Dr, Bethesda, MD 20892 USA; 2 Office of Biostatistics Research, National Heart, Lung, and Blood Institute, National Institutes of Health, Bethesda, MD USA; 3 Laboratory of Epidemiology and Population Science, National Institute on Aging, National Institutes of Health, Baltimore, MD USA

**Keywords:** Risk prediction, Cardiovascular disease, Social determinants of health

## Abstract

**Background:**

The Predicting Risk of Cardiovascular Disease EVENTs (PREVENT) equations were designed to predict CVD outcomes in adults aged 30–79, using a race-free model that includes kidney function and statin use. Their performance compared to the Pooled Cohort Equations (PCE) needs evaluation in diverse populations to assess clinical utility.

**Methods:**

In a sample of Black and White individuals aged 30–64 from the Healthy Aging in Neighborhoods of Diversity across the Life Span (HANDLS) cohort, we assessed the performance of the base PREVENT equations and the PCE using complementary methods (discrimination, reclassification) and evaluated if adding social determinants of health (SDoH) to base PREVENT improved the prediction of CVD deaths.

**Results:**

Among 1,998 HANDLS participants free of CVD, the 10-year cumulative incidence of CVD death (95% confidence intervals [CI]) was 2.75% (2.10%, 3.54%). The PREVENT equation exhibited better discrimination (AUC_t=10_ (95% CI): 0.77 (0.71, 0.83)) than the PCE (0.71 (0.64, 0.78)), and modest improvement in risk reclassification (continuous net reclassification index: 0.38 (0.10, 0.67)). Out of four SDoH variables (poverty, education, employment, and homeownership status) evaluated, only unemployment was strongly associated with CVD mortality independently of PREVENT-predicted risk (HR (95% CI): 3.39 (1.80, 6.40)). However, updating PREVENT-predicted risks with unemployment status only modestly improved discrimination and risk reclassification.

**Conclusions:**

In a socially and racially diverse cohort, the PREVENT total CVD risk equation demonstrated better discrimination and risk reclassification compared to the PCE. After adjustment for PREVENT-estimated risk, unemployment remained strongly associated with CVD mortality, but only modestly, and non-significantly, improved model discrimination and risk reclassification.

**Supplementary Information:**

The online version contains supplementary material available at https://doi.org/10.1186/s12889-026-27358-5.

## Introduction

Evaluating cardiovascular disease (CVD) risk in disease-free adults is key to primary prevention, and clinical guidelines from the American College of Cardiology/American Heart Association (ACC/AHA) recommend using risk prediction equations for this purpose [1]. Over the past several decades, several risk equations have been created, including the sex- and race-stratified Pooled Cohort Equations (PCE) [2] for predicting 10-year risk of atherosclerotic CVD (ASCVD), endorsed in the 2019 ACC/AHA Guideline on the Primary Prevention of Cardiovascular Disease for adults ages 40-79 [1]. These scores are designed to be calculated and implemented at point of care [3]. Motivated by several considerations including the high burden of cardiovascular-kidney-metabolic (CKM) syndrome and the goal of removing race from clinical algorithms [4], the AHA recently developed sex-specific and race-free risk equations (Predicting Risk of CVD Events [PREVENT]) to predict CVD in adults aged 30–79 years based on traditional clinical risk factors [5]. An “enhanced” version of the PREVENT model also includes area-level social factors [4, 5], as measured by Social Deprivation Index (SDI) based on ZIP code [6, 7].

The derivation and validation populations for PREVENT included mostly (80%) self-identified White participants, and the enhanced PREVENT models including SDI were developed in a smaller subset of participants with available measures [5]. Thus, given the varied distributions of CVD risk factors [8], prevalence, and mortality [9] across different demographic groups, further evaluation of the performance of the PREVENT equations in different populations is necessary to fully ascertain their utility. Additionally, determining whether the inclusion of individual-level social determinants of health (SDoH) variables improves CVD risk prediction in diverse populations is an ongoing area of research [10–12] particularly since the relationships between SDoH and CVD outcomes have been observed to vary across populations [13].

The Healthy Aging in Neighborhoods of Diversity across the Life Span (HANDLS) cohort offers a unique opportunity to evaluate the base PREVENT equations and incorporation of SDoH, as it is a biracial cohort of community-dwelling Black and White adults across a range of socioeconomic status [14]. Therefore, our goal was to leverage the characteristics of HANDLS to compare the performance of the PCE and PREVENT and assess if adding SDoH variables would improve the performance of PREVENT. Recognizing that there is not one single method to evaluate the model performance such that risk prediction is inherently multi-dimensional [15], we evaluated multiple measures of model performance, including discrimination and risk reclassification.

## Methods

### Cohort description

HANDLS is a prospective population-based cohort study designed to examine how socioeconomic status and race impact the development of age-related health disparities [14]. Black and White participants aged 30–64 years old in Baltimore City were recruited in a four-way factorial cross of sex, age, race, and poverty status (defined by self-reported household incomes above/below 125% of the 2004 Federal Poverty Guidelines) between 2004 and 2009 [14]. Race was self-identified at recruitment. Participants were not eligible for recruitment if they were currently pregnant, within 6 months of cancer treatment, had AIDS, were unable to provide written informed consent, or lacked verifiable identification. There were 3,720 eligible participants identified who were invited to participate in the baseline study visit (occurring between 2004 and 2009), approximately 75% of whom ultimately completed the baseline visits [14]. As described in the original HANDLS study design manuscript, race, sex, and age, but not poverty status, were associated with the probability of completing the HANDLS examination [14]. At baseline exams, physical examinations, medical and social histories, including various social determinants of health variables, and collection of biospecimens were performed in mobile research vehicles [14]. All participants gave written informed consent. The HANDLS study was approved by the National Institute of Environmental Health Sciences Institutional Review Board.

### Ascertainment of PCE and PREVENT predictor variables

The PCEs are sex and race-specific and estimate risk of ASCVD using age, systolic blood pressure (SBP), total cholesterol, HDL cholesterol (HDL-C), diabetes, current smoking status, and anti-hypertensive medication use [2]. In this study we focused on the base PREVENT equations for estimating total CVD (encompassing both ASCVD and heart failure), which are sex-specific but race-free, and include all of the PCE predictors plus estimated glomerular filtration rate (eGFR) and statin medication use [5]. These predictor variables were measured in HANDLS by clinical laboratory assessments, physical examination, or medical history [16]. To align with the approach used in the development of the PREVENT equations [5], we included fasting and non-fasting patients, and calculated the race-free estimate of eGFR from serum creatinine using the CKD-Epi 2021 [17] Eqs. [18, 19]. For 4.9% (*n* = 98) of our final primary analysis sample, serum creatinine was measured by the National Institute on Aging Clinical Research Branch Core Laboratory using a modified kinetic Jaffe method (CREA method, Dade Dimension X-Pand Clinical Chemistry System, Siemens Healthcare Diagnostics Inc., Nework, DE) and for the remainder of participants, serum creatinine was measured at Quest Diagnostics, Inc. by isotope dilution mass spectrometry (Olympus America Inc., Melville, NY) and standardized to the reference laboratory at the Cleveland Clinic. SBP was measured once in each arm, and we averaged these measurements; if only one measurement was available, this single value was used.

### Study population and exclusions

**Fig. 1:**
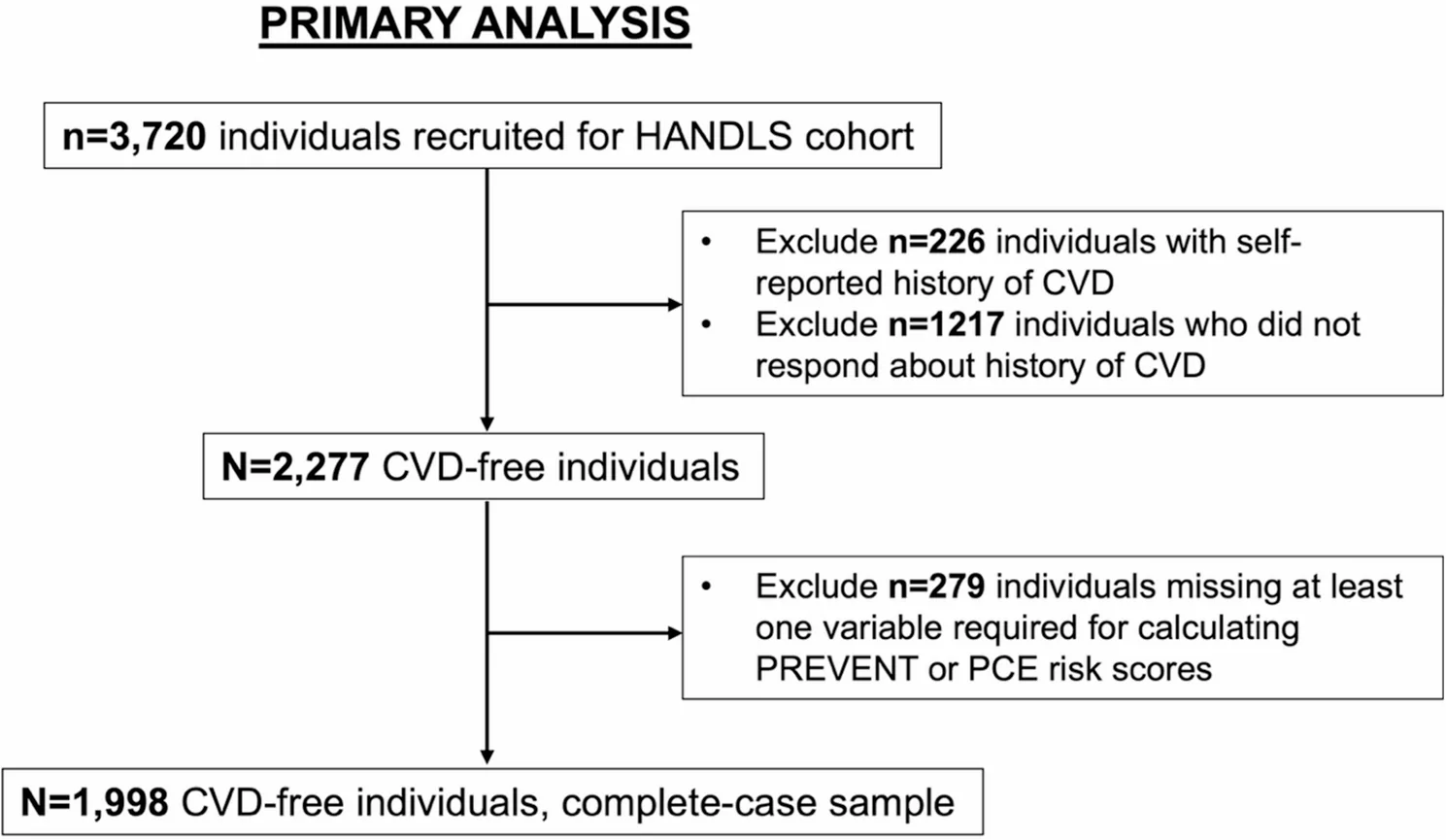
Flow diagram of inclusion criteria for primary complete-case analysis sample from the HANDLS cohort

Figure 1 presents a flow diagram of the exclusions we applied to assemble our final primary analysis study sample. We excluded HANDLS participants with self-reported history of coronary artery disease, stroke, heart failure, or myocardial infarction (*n* = 226) as well as those who were missing self-reported medical history data (*n* = 1217). We subsequently excluded any participants who were missing relevant PCE and PREVENT predictor variables (*n* = 279), consistent with the complete-case approach used in the derivation of the PCE [2] and PREVENT [5] equations.

### Calculation of PREVENT and PCE 10-year CVD risk estimates

We used the “CVrisk” R package (version 1.1.1) code to calculate the PCE 10-year ASCVD risk estimates [20]. We used the reported coefficients from the base PREVENT equations for 10-year total CVD risk [5] to calculate PREVENT 10-year total CVD risk estimates. The PREVENT + SDI equation requires ZIP code, which was unavailable in our data. Since the PCE were designed for participants ages 40–79 but PREVENT was designed for participants ages 30–79 [2, 5], we winsorized any participant with age < 40 years to 40 years for the computation of the PCE risk estimates in order to compare the same populations for PCE and PREVENT. Furthermore, both PCE and PREVENT were derived in populations in which participants with extreme values for SBP, cholesterol, and BMI were excluded [2, 5]. However, to better reflect how individuals with clinical values outside these ranges would be assessed in clinical practice, for participants with values above or below the PREVENT exclusion criteria [5] we winsorized values to the maximum and minimum exclusion thresholds to compute their estimated risks.

### Sensitivity analyses samples

We also assembled four additional sensitivity analysis samples in which to evaluate PREVENT and PCE. First, because PCE was originally developed for individuals ages 40–79, we excluded all individuals < 40 years old from our primary analysis population (*n* = 422) (“sensitivity analysis A” sample, flow diagram shown in Figure S1). Second (“sensitivity analysis B” sample, flow diagram shown in Figure S2), we excluded individuals with self-reported history of CVD (*n* = 226) but did *not* exclude individuals who were missing self-reported CVD medical history. Although many of these individuals were subsequently excluded upon removing any participants missing relevant PCE and PREVENT predictor variables (*n* = 1400), those with complete predictor variables remained, resulting in a slightly larger size than the primary analysis population. Therefore, this sensitivity sample assumed that individuals who otherwise completed relevant baseline examination procedures (yielding data for PREVENT/PCE calculations) but who did not report on CVD medical history were likely to be CVD-free.

Finally, due to possible differences in creatinine measurements across the two laboratory sites (since values measured at Quest laboratories were the only ones that were standardized), we excluded the *n* = 236 individuals whose creatinine was not measured at a Quest laboratory. We then applied the same exclusion procedures as our primary analysis sample (“sensitivity analysis C” sample, flow diagram shown in Figure S3) and in sensitivity analysis B described above (“sensitivity analysis D” sample, flow diagram shown in Figure S4), and carried out all statistical analyses in these samples.

### Outcome assessment

The PCE and PREVENT equations were designed to estimate risk of composite fatal and non-fatal cardiovascular events [2, 5]. However, due to missing data and three to five years between visits for self-reported medical history, causing significant interval censoring, we focused only on CVD deaths, which were obtained from the National Death Index from 2004 to 2021. CVD deaths were defined by ICD-10 codes I00-I78; all other deaths were considered non-cardiovascular for competing risk models. We estimated the 10-year cumulative incidence of CVD death, accounting for competing risk of non-CVD deaths [21].

### SDoH variables

The SDI is defined based on seven variables capturing area-level poverty, education, employment, homeownership, home overcrowding, single family households, and vehicle access [6, 7]. We were unable to link to area-level SDI in HANDLS, but individual-level SDoH variables were ascertained in HANDLS via self-report on social history questionnaires. Therefore, we instead focused on available individual-level SDoH variables reflecting the domains included in the area-level SDI: poverty status (above versus below federal poverty delimiter), education (self-reported attainment of less than high school education versus high school or more), self-reported home ownership status (renting/other versus owning home), and self-reported employment status within the last month, at the time of baseline study examination (unemployed versus employed). For individuals who reported unemployment, information was also collected on their stated reasons for unemployment, with the questionnaire response options: “Taking care of house/kids,” or other self-described other caretaking, “student,” “disabled” or other self-described health-related reasons, “retired,” “can’t find a job,” “doesn’t need or want to work,” “other.” For conceptual consistency with the SDI area-level housing variable, participants who responded to the housing questionnaire with “home owned or rented by friend or relative,” “rent your home,” or “other” were collapsed into a single category (“Rent or other”) and compared with those who reported owning their home.

### Statistical analysis: Evaluating performance of PREVENT and PCE equations

Because the lack of non-fatal outcome data precludes the assessment of the composite outcome for which PREVENT and PCE were designed, we restricted our model evaluation to assessing the discriminative ability of the risk scores for CVD mortality, and continuous net risk reclassification. Thus, this study is an assessment of prognostic performance but not a full formal model validation.

### Discrimination

We evaluated discrimination by calculating the time-dependent cumulative/dynamic area under the receiver operating characteristic curve (AUC_t_) and standard error, accounting for competing risks, at 10 years, where controls were defined as any individual *i* that was not a case, with T_follow−up_ > *t =* 10 years, or with T_follow−up_ ≤ *t* = 10 years and a non-CVD death [22, 23] and the calculated risk estimates (PCE, PREVENT) were evaluated as the “marker” of interest. Discrimination was evaluated overall, and stratified by race.

### Risk reclassification

Prior to evaluating risk reclassification and model updating, we recalibrated the PCE and PREVENT risk estimates using the linear predictor method from Steyerberg [24]. We used the estimated natural log(-log(S(t_10_)) and natural log-odds of the 10-year year risk from the PCE and PREVENT equations, respectively, as a single linear predictor in a cause-specific Cox proportional hazards model with follow-up time administratively censored at 10-years, CVD mortality as the outcome and non-CVD deaths as competing risk, to predict 10-year absolute risk. We evaluated change in risk classification between the recalibrated PCE and PREVENT risk estimates by calculating the continuous net reclassification improvement (NRI) by 10 years as described by Pencina et al. [25]. We adapted this to the competing risks context by estimating cause-specific cumulative incidence at 10 years for observed event rates [21], and used the percentile bootstrap method to calculate 95% confidence intervals (CI) [26].

### Updating risk with SDoH variables

Because we were unable to evaluate the enhanced PREVENT risk equation that includes area-level SDI, we sought to determine whether updating the base PREVENT equations with individual-level SDoH variables (poverty status, education, homeownership, and employment status) improved model performance. We first evaluated associations between each of these SDoH variables and CVD mortality, estimating cause-specific Cox proportional hazards models and administratively censoring follow-up at 10 years. We adjusted for age and sex (model 1) and for the log-odds of the recalibrated PREVENT risk estimate (model 2). The proportional hazards assumption was assessed by plotting Schoenfeld residuals [27, 28].

SDoH variables that remained statistically significant after adjustment for the log-odds of the recalibrated PREVENT risk were carried forward to evaluate model performance after updating the base PREVENT equations with a given individual-level SDoH. To update the PREVENT equations, we adapted the approach described by Kooter et al. [29] and Hageman et al. [30], which updates individual predicted risks by accounting for both the effect of a given dichotomous predictor and its population prevalence. We used the formulas provided in the supplemental materials of Hageman et al. [30] to calculate the updated risks, but aligning with Kooter et al.’s original approach [29], applied relative risks instead of subdistribution hazards in the calculations. A worked example of our calculations, adapting Hagemen et al.’s approach [30], is provided in Supplementary Table 1. After updating the risk estimates, we assessed discrimination, also stratifying by the SDoH variable of interest, and risk reclassification.

All analyses were performed using R statistical software, version 4.4.2 (R Core Team, Vienna, Austria) [31] using the packages nephro, [18, 19] CVrisk [20], riskRegression [32, 33], timeROC [23] cmprsk [21], nricens [26], tidycmrsk [34], and survival [27, 28].

## Results

### Description of study population

After excluding participants with documented prevalent CVD or missing at least one relevant PCE or base PREVENT risk equation variable, our final primary analysis study population included 1,998 individuals. Individuals who were excluded from the analysis due to missing data were slightly younger, more likely to be Black, more likely to be below the federal poverty delimiter, and exhibited a slightly higher 10-year cumulative incidence of CVD mortality than the individuals included in the complete-case analysis sample (Table 1). However, the PREVENT and PCE risk score-related variables, when available, were largely similar across both samples. The primary analysis sample (Table 1) differed from the original PREVENT derivation population in several ways. 56% of HANDLS participants self-identified as Black, compared to 9% in the original PREVENT population [5]. HANDLS participants were younger (mean age 48 years (SD = 9)) than the PREVENT population (mean age 53 (13) years) but had a higher diabetes prevalence (16% vs. 11%) [5]. The prevalence of current cigarette smoking was markedly higher in HANDLS (48%) compared to the PREVENT population (6%) [5].

**Table 1:** Demographic characteristics, PREVENT/PCE risk score variables, and social determinants of health variables in the HANDLS population

Variable	Complete-case primary analysis sample (*n* = 1998)	Individuals excluded from complete-case analysis sample due to missing self-reported history of CVD or missing risk score variables (total *n* = 1496)
10-year cumulative incidence of CVD death (95% CI)	2.75% (2.10%, 3.54%)	3.54% (2.69%, 4.57%)
Age, years	48 (9)	47 (10)
Black	1,122 (56%)	931 (62%)
White	876 (44%)	565 (38%)
Female	1,124 (56%)	780 (52%)
PREVENT and PCE risk score-related variables		
BMI, kg/m^2^	30 (8)	29 (8)
Missing	0	867
Total cholesterol, mg/dL	188 (43)	187 (44)
Missing	0	959
HDL cholesterol, mg/dL	53 (17)	55 (19)
Missing	0	960
Systolic blood pressure, mmHg	120 (18)	120 (18)
Missing	0	939
eGFR, mL/min/1.73m^2^	91 (18)	83 (22)
Missing	0	1,052
Current cigarette smoker	951 (48%)	197 (54%)
Missing	0	1,134
Diabetes	310 (16%)	87 (16%)
Missing	0	961
Use of antihypertensive medication	578 (29%)	107 (24%)
Missing	0	1,042
Use of lipid-lowering medication	227 (11%)	19 (4.2%)
Missing	0	1,040
Social Determinants of Health		
*Poverty Status*		
Below 125% of the 2004 Federal Poverty Guidelines level	788 (39%)	636 (43%)
*Education*		
Less than high school education	608 (31%)	544 (37%)
Missing	37	29
*Homeownership*		
Owns own home	829 (43%)	499 (34%)
Rents home	823 (42%)	744 (51%)
Home owned or rented by friend or relative	289 (15%)	217 (15%)
Other	6 (0.3%)	3 (0.2%)
Missing	51	33
*Employment – dichotomous*		
Unemployed within the last month	776 (40%)	634 (43%)
Employed within the last month	1171 (60%)	829 (57%)
Missing	51	33
*Three levels of unemployment*		
Voluntary unemployment/other	217 (27%)	160 (25%)
Involuntary unemployment, can’t find job	181 (23%)	130 (21%)
Involuntary unemployment, disabled/health-related reasons	378 (49%)	344 (54%)

Continuous variables are reported as mean (standard deviation)

Categorical variables are reported as n (%)

For variables with missing data, percentages are reported as a percent of the complete-case sample

Voluntary unemployment/other includes individuals who reported the following reasons for unemployment: Taking care of house/kids or other caretaking; Doesn’t need or want to work; Student; Retired; Other

Additionally, 39% of HANDLS participants were below 125% of the federal poverty level, 31% had less than a high school education, and 40% were unemployed, more than half of whom stated their reasons for being unemployed as due to the “involuntary” reasons of disability or health-related reasons, or an inability to find a job. Within 10-years of follow-up, 218 deaths occurred, with neoplasms (C00-D49, *n* = 62) accounting for the highest number of deaths from a single ICD-10 grouping category [35], followed by CVD-related deaths (I00-I78, *n* = 55). The 10-year cumulative incidence (95% CI) of CVD deaths, accounting for competing risks of non-CVD deaths, was 2.75% (2.10%, 3.54%). These summary demographic characteristics were generally consistent across all four sensitivity analysis samples (sensitivity sample B: *n* = 2094, sensitivity sample C: *n* = 1900, sensitivity sample D: *n* = 1992), though the cumulative incidence of CVD was highest in the “sensitivity analysis A” sample excluding individuals < 40 years (3.43% (2.61%, 4.41%)) (Supplementary Tables 2, 3, 4, 5).

### Discrimination and risk reclassification

Discrimination, as measured by AUC_t=10_ (95% CI), was significantly higher for PREVENT than PCE in the primary analysis sample (PREVENT: 0.77 (0.71, 0.83), PCE: 0.71 (0.64, 0.78), *p* = 0.01) (Table 2). This was consistent across race, with race-stratified AUC_t=10_ estimates for PCE and PREVENT similar to the PCE and PREVENT estimates in the total sample. The continuous NRI (95% CI) comparing the PREVENT equations to PCE was modest at 0.38 (0.10, 0.67), with a greater reclassification among non-events (0.29 (0.25, 0.33)) than events (0.09 (-0.18, 0.38)) (Table 2). When we excluded individuals less than 40 years old (sensitivity analysis A sample, *n* = 1576), PREVENT also exhibited higher discrimination and NRI compared to the PCE, though the NRI and AUC_t=10_ estimates were numerically lower for both models than in the primary analysis sample (PCE: AUC_t=10_ (95% CI) = 0.67 (0.59, 0.75), PREVENT: AUC_t=10_ (95% CI) = 0.73 (0.66, 0.80)) (Supplementary Table 6). AUC_t=10_ and NRI estimates in the other sensitivity analysis samples were very similar to the results in our primary analysis sample, though NRI values were numerically lower in the smaller Quest-restricted samples (Supplementary Tables 7, 8, 9).

**Table 2:** Discrimination of PCE and PREVENT in HANDLS, primary analysis sample

Total population				
	PCE		PREVENT	
*N (CVD deaths by 10 years)*	*AUC*_*t=10*_ *(95% CI)*			*p-value*
1998 (55)	0.71 (0.64, 0.78)		0.77 (0.71, 0.83)	0.01
	*Continuous NRI PREVENT vs. PCE*			
	*NRI (95% CI)*	*NRI event (95% CI)*	*NRI non-event (95% CI)*	
	0.38 (0.10, 0.67)	0.09 (-0.18, 0.38)	0.29 (0.25, 0.33)	
*Black persons*				
*N (CVD deaths by 10 years)*	*AUC*_*t=10*_ *(95% CI)*			*p-value*
1122 (37)	0.70 (0.61, 0.80)		0.77 (0.69, 0.85)	0.02
*White persons*				
*N (CVD deaths by 10 years)*	*AUC*_*t=10*_ *(95% CI)*			*p-value*
876 (18)	0.71 (0.59, 0.83)		0.76 (0.66, 0.86)	0.14

The reported AUC_t=10_ is the AUC_2 value reported in the timeROC() R package

### Evaluating associations between SDoH variables and CVD mortality

**Fig. 2:**
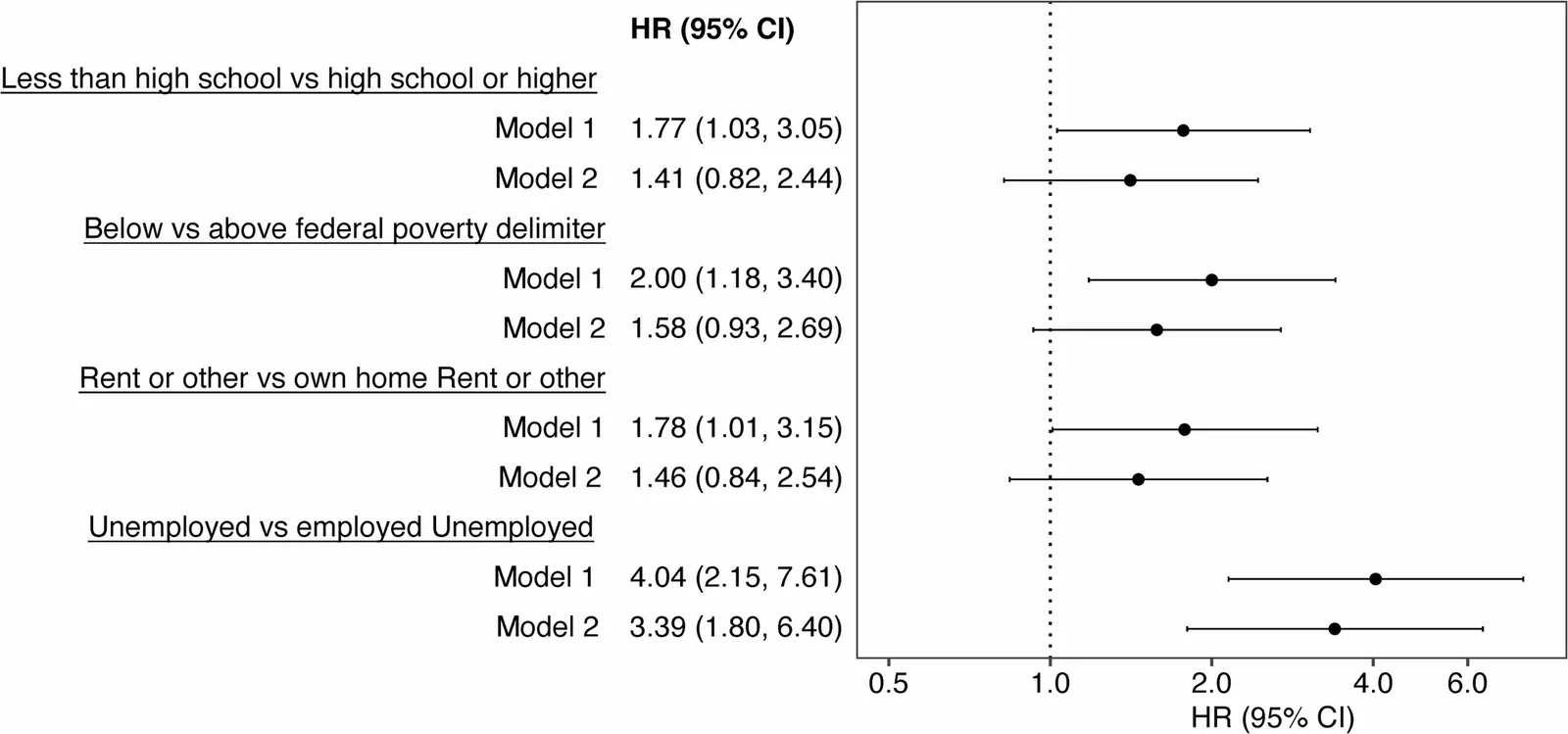
Hazard ratios (HR) (95% confidence intervals) for associations between select individual-level social determinants of health variables and CVD mortality in HANDLS primary analysis sample. Model 1: adjusted for age and sex as reported at the baseline visit. Model 2: adjusted for the log-odds of the recalibrated PREVENT risk estimate. HR (95% CI) are plotted on the log-scale

After adjusting for age and sex, all four individual-level SDoH variables were positively significantly associated with CVD mortality (Fig. 2). After adjusting for the PREVENT risk estimate, only employment status remained significantly associated with CVD mortality (HR (95% CI) = 3.39 (1.80, 6.40), *p* = 1.7E-04). These results were generally consistent in sensitivity analysis samples A and B and in the Quest laboratory-restricted sensitivity samples (Figures S5, S6, S7, S8). Because the prevalence of smoking was particularly high among those who were unemployed (58.8%) compared to those who were employed (40%) we also assessed for interaction between unemployment and smoking status, but this was not statistically significant (*p* = 0.15).

### Updating base PREVENT risk estimates with employment status data

Discrimination of PREVENT was somewhat lower among those who were unemployed (AUC_t=10_ (95% CI) = 0.73 (0.65, 0.81)) than those who were employed (AUC_t=10_ (95% CI) = 0.75 (0.65, 0.86)) (Table 3). After updating the PREVENT risks with unemployment status, discrimination was slightly better than PREVENT alone (PREVENT+Unemployment: AUC_t=10_ (95% CI) = 0.79 (0.74, 0.85); PREVENT alone (0.76 (0.70, 0.83)) though this difference was not statistically significant (*p* = 0.12) (Table 3). These results were also consistent across race strata. Continuous NRI (95% CI) of the updated PREVENT+Unemployment estimates compared with PREVENT was 0.73 (0.46, 0.97), with greater reclassification of events (0.51 (0.25, 0.74)) than non-events (0.22 (0.18, 0.27)). In sensitivity analysis sample A, we observed a similar non-significant increase in discrimination for PREVENT+Unemployment than PREVENT alone, though AUC_t=10_ estimates were numerically lower for both models than in the primary analysis sample, and AUC_t=10_ was slightly worse in employed persons than unemployed persons, but we note that the confidence intervals overlap (Supplementary Table 10). We also observed similar results to the primary analysis in sensitivity analysis samples B, C, and D (Supplementary Tables 11, 12, 13).

**Table 3:** Discrimination of PREVENT and updated PREVENT + Unemployment in HANDLS primary analysis sample, *n* = 1947 with complete employment data

Total population				
	PREVENT	PREVENT+Unemployment		
* N (CVD deaths by 10 years)*	*AUC*_*t=10*_ *(95% CI)*			*p-value*
1947 (53)	0.76 (0.70, 0.83)	0.79 (0.74, 0.85)		0.12
	*Continuous NRI PREVENT vs. PCE*			
	*NRI (95% CI)*	*NRI event (95% CI)*	*NRI non-event (95% CI)*	
	0.73 (0.46, 0.97)	0.51 (0.25, 0.74)	0.22 (0.18, 0.27)	
*Black persons*				
* N (CVD deaths by 10 years)*	*AUC*_*t=10*_ *(95% CI)*			*p-value*
1117 (37)	0.77 (0.69, 0.85)	0.79 (0.72, 0.86)		0.29
*White persons*				
* N (CVD deaths by 10 years)*	*AUC*_*t=10*_ *(95% CI)*			*p-value*
830 (16)	0.75 (0.64, 0.86)	0.79 (0.68, 0.89)		0.27
*Unemployed persons*				
* N (CVD deaths by 10 years)*	*AUC*_*t=10*_ *(95% CI)*			*p-value*
776 (40)	0.73 (0.65, 0.81)			--
*Employed persons*				
* N (CVD deaths by 10 years)*	*AUC*_*t=10*_ *(95% CI)*			*p-value*
1171 (13)	0.75 (0.65, 0.86)			--

The population RR used for PREVENT+Unemployment model updating was 2.46 (see Supplementary Table 1 for calculation)

The reported AUCt=10 is the AUC_2 value reported in the timeROC() R package

## Discussion

Among 1,998 middle-aged White and Black participants across the socioeconomic spectrum in the HANDLS cohort, the PREVENT total CVD 10-year risk equation exhibited improved discrimination and modestly improved net reclassification compared to the PCE for CVD mortality risk prediction. These results were consistent across several sensitivity analysis samples, suggesting these findings are robust in this HANDLS population. Unemployment was strongly associated with CVD mortality independently of PREVENT, suggesting that residual risks related to unemployment are potentially important.

### Comparing PCE and PREVENT

Our results are largely consistent with prior comparisons of the PCE and PREVENT models [5]. In validation datasets, PREVENT exhibited modestly improved discrimination and calibration compared to PCE overall, and across racial groups [5]. In HANDLS, discrimination was about 6% greater for PREVENT than PCE, and this was consistent across race. PREVENT also exhibited higher discrimination and improved risk reclassification compared to PCE in sensitivity analyses excluding individuals < 40 years old, though both models had lower discrimination in this older population than in the full primary analysis sample. The lack of differences in PREVENT or PCE across race is consistent with recent results from Ghosh et al. which showed that race-free versions of the PCE yielded similar discrimination and calibration as the original race-specific models when applied in the biracial Racial Differences in Stroke Study (REGARDS) cohort [10]. These results give support to the movement towards race-free risk prediction models [36].

Removal of race from the PREVENT prediction models was motivated by the argument that race impacts health secondarily through measured and unmeasured SDoH [37–39]. Multiple previous studies have linked adverse SDoH, including employment status, income, and education, with increased CVD risk [13, 40], though the strength and significance of these associations have varied by race and ethnicity [13]. Therefore, assessing whether the inclusion of specific SDoH variables instead of race as a proxy improves CVD risk prediction models is of great interest [40]. Indeed, this was a motivating factor for the development of the PREVENT and PREVENT + SDI risk equations [4, 5], though the addition of SDI to the base PREVENT model yielded very minimal improvements in discrimination and similar calibration to the model without SDI [5].

### Adding SDoH to CVD risk prediction models

Recent literature on the extent to which SDoH variables improve CVD risk prediction is inconclusive [10–12]. For example, adding several area- and individual-level SDoH variables to Ghosh et al.’s rederived race-free version of the PCE did not improve model performance in REGARDS [10], aligning with findings in the MESA cohort that individual-level social disadvantage variables did not improve the performance of the PCE [11] or the MESA Risk Score [12]. Conversely, in a different analysis of the REGARDS cohort, risk reclassification was slightly improved when annual household income or a cumulative number of indicators of deprivation were included in the model [41]. Another recent study in several diverse cohorts reported that adding individual and area-level SDoH to the PCE modestly improved discrimination and calibration among Black participants, and adding individual-level SDoH to the PREVENT + SDI equations also modestly improved calibration [42]. Thus, our study adds to the growing body of literature suggesting only minimal-to-modest effects of SDoH on improving CVD risk prediction. Although unemployment was strongly associated with incident CVD mortality, updating PREVENT with unemployment status only modestly increased discrimination and risk reclassification.

It has been hypothesized that the modest impact of SDoH on risk score performance, despite strong associations between SDoH and CVD, is due to the more “upstream” effects of SDoH on the cardiovascular risk factors included in risk Eqs. [12, 38, 39]. It is also conceivable that current measures of SDoH do not capture the inherent complexity of these factors [38]. Ongoing efforts to collect better data on SDoH in clinical settings [37, 43] should improve coverage and granularity, leading to the development of better metrics, such as SDoH risk scores [13], which may in turn help improve CVD risk prediction [40].

### Limitations

Some limitations must be considered. The high levels of missingness and resulting small sample sizes in the HANDLS cohort is an important limitation, limiting precision for estimates and power for assessing model updating. The individuals who were excluded reflect a population with a higher cumulative incidence of CVD mortality and slightly more social deprivation. Therefore, had they been included in our analysis, it is possible that stronger associations between SDoH and CVD mortality may have been detected. However, we acknowledge that the performance of PREVENT and PCE in the complete-case sample may not totally reflect how these risk equations would perform in this population.

Additionally, we were only able to assess total CVD mortality, not the composite CVD outcome for which PCE and PREVENT were originally developed. As a result, this analysis only focused on evaluating discrimination and risk reclassification, but not calibration. Due to the follow-up period, we only evaluated the 10-year total CVD equations. Furthermore, due to lack of zip code information to link to area-level SDI, we were unable to evaluate the PREVENT + SDI model and compare its performance (discrimination, risk reclassification) to the base PREVENT and PREVENT+Employment models. Finally, we recognize that dichotomizing employment status ignores different potential reasons for unemployment (e.g. voluntary versus involuntary unemployment), which may confer different CVD risks. However, the limited sample and event size precluded robust association analyses with a more granular employment status definition.

## Conclusions

In a socioeconomically and racially diverse cohort, the PREVENT total CVD risk equation demonstrated superior discrimination and risk reclassification compared to the PCE in predicting cardiovascular mortality. Unemployment remained strongly and independently associated with cardiovascular mortality even after adjustment for PREVENT-estimated risk. However, this only resulted in very modest, non-significant improvements in model discrimination, and modest risk reclassification. Future studies with larger sample sizes will be necessary to determine whether incorporation of unemployment status substantially improves risk prediction and thus justifies its collection in clinical practice.

## Supplementary Information

Supplementary Material 1.

## Data Availability

Public data sharing is not permitted due to ethical restrictions. Qualified researchers can request data sharing through a Data Use Sharing Agreement as described at https://handls.nih.gov/06Coll.htm.

## Abbreviations

PREVENT: Predicting Risk of Cardiovascular Disease Events
PCE: Pooled Cohort Equations
HANDLS: Healthy Aging in Neighborhoods of Diversity across the Life Span
CVD: Cardiovascular disease
CKM: Cardiovascular-kidney-metabolic syndrome
AUC_t_: time-dependent area under the curve
ACC/AHA: American College of Cardiology/American Heart Association
SDoH: social determinants of health
SDI: Social Deprivation Index
NRI: net reclassification improvement
