# Supplementary material for: Prognostic value of PREVENT, PCE, and social determinants of health for cardiovascular mortality in the Healthy Aging in Neighborhoods of Diversity across the Life Span (HANDLS) study
Source: BMC Public Health. 2026 Apr 29;26:1885. doi: 10.1186/s12889-026-27358-5 (PMC13274012; doi:10.1186/s12889-026-27358-5)
Supplement: Supplementary file 1 — Supplementary Material 1. [file 12889_2026_27358_MOESM1_ESM.docx]

Table of Contents

[Flow Diagrams for Sensitivity Analysis Samples 2](#_Toc225494487)

[Calculation example for updating risk scores based on SDoH variable 4](#_Toc225494488)

[Table 1 Demographic Characteristics for Sensitivity Analysis Samples 5](#_Toc225494489)

[Discrimination and risk reclassification comparing PREVENT and PCE in Sensitivity Analysis Samples 9](#_Toc225494490)

[Forest plots for SDoH associations with CVD mortality in Sensitivity Analysis Samples 11](#_Toc225494491)

[Discrimination and risk reclassification for PREVENT+Unemployment updating versus PREVENT in Sensitivity Analysis Samples 15](#_Toc225494492)

# Flow Diagrams for Sensitivity Analysis Samples


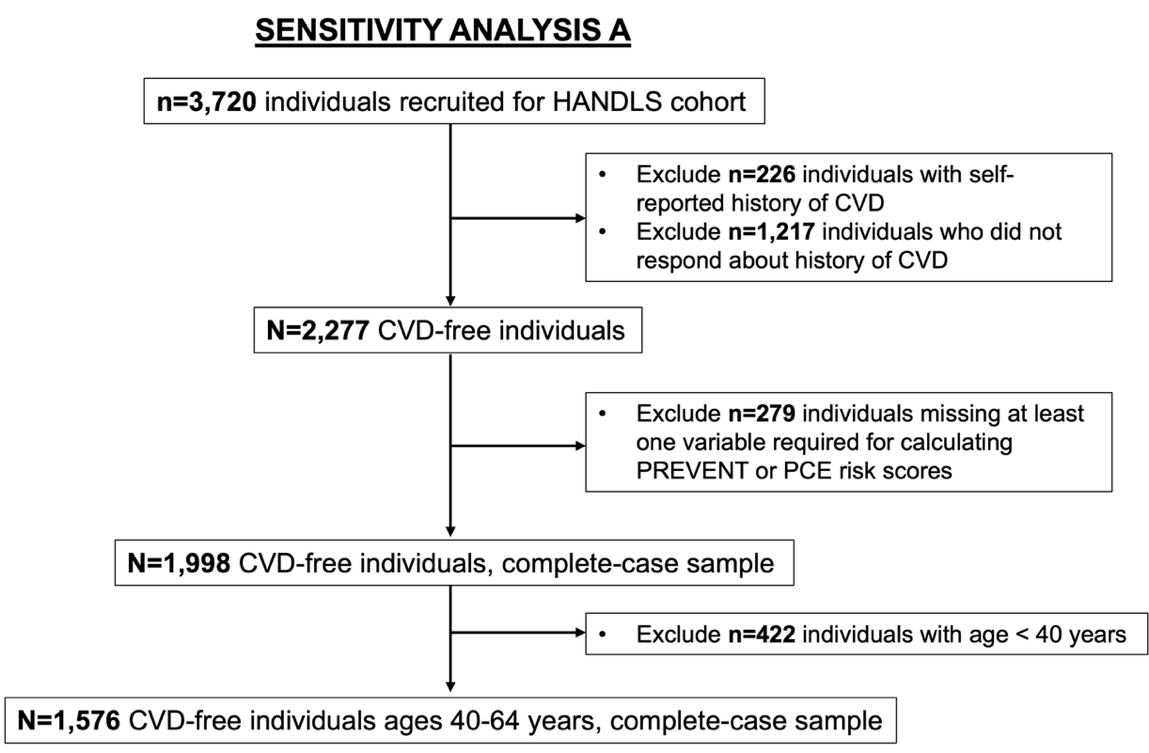


**Figure S1. Flow diagram for sensitivity analysis A sample, excluding individuals < 40 years of age from primary analysis sample.**


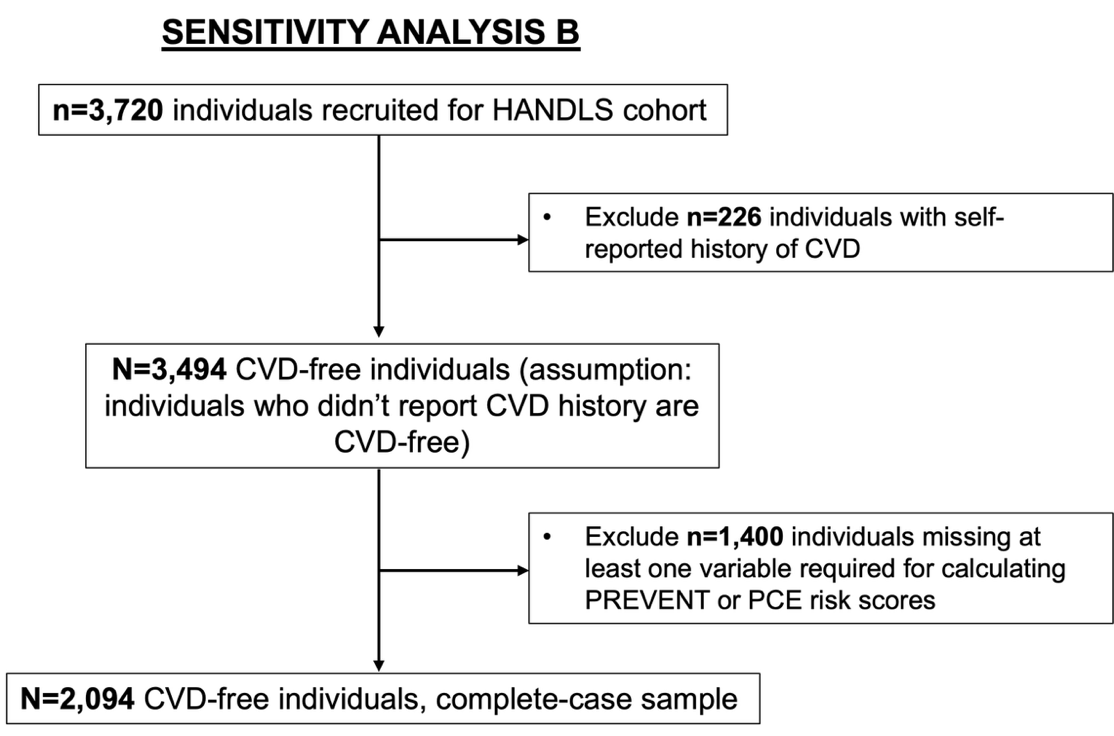


**Figure S2. Flow diagram for sensitivity analysis B sample, complete-case sample assuming individuals missing CVD medical history did not have prevalent CVD.**


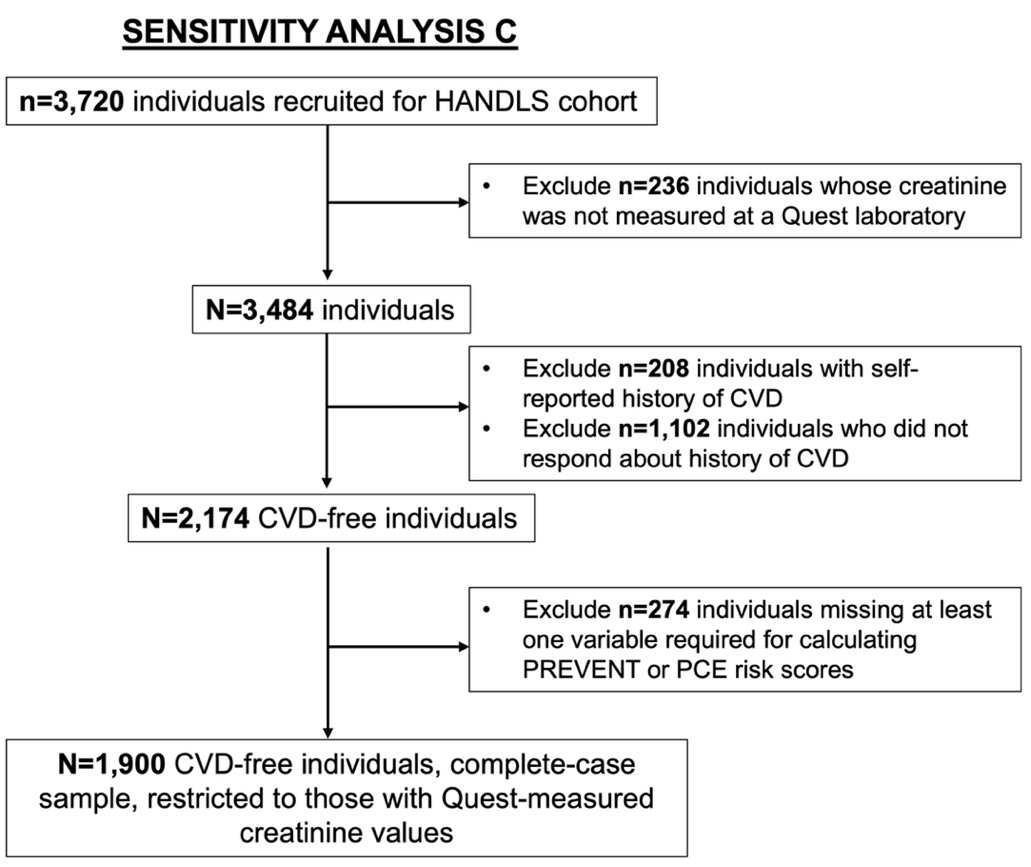


**Figure S3. Flow diagram for sensitivity analysis C sample, complete-case sample, restricted to individuals with creatinine measured from Quest laboratories.**


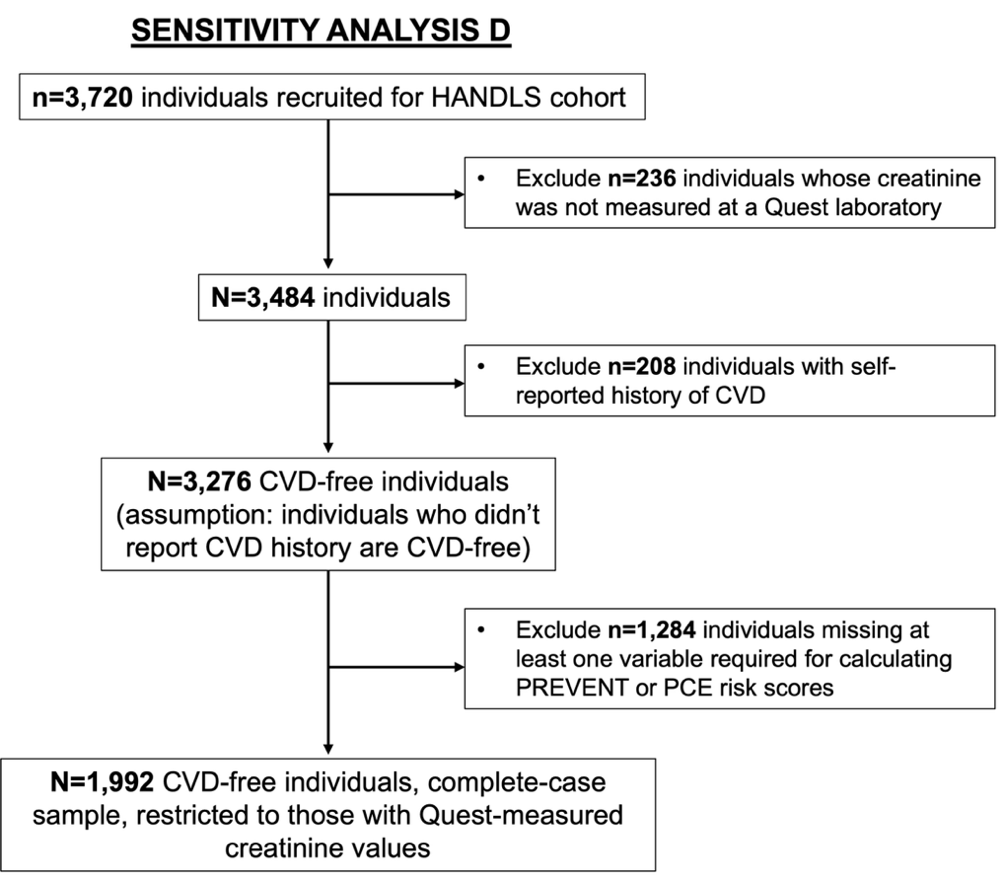


**Figure S4. Flow diagram for sensitivity analysis D sample, complete-case sample assuming individuals missing CVD history data were CVD-free, restricted to individuals with creatinine measured from Quest laboratories.**

# Calculation example for updating risk scores based on SDoH variable

| **Supplementary Table 1. Toy example of calculation of updated risk scores based on SDoH variable** | | | |
| --- | --- | --- | --- |
| **Step 1: Calculate PREVENT base 10-year CVD risk** | | | |
| Age (years) | 45 | |  |
| Sex | Female | |  |
| SBP (mmHg) | 120 | |  |
| eGFR (mL/min/1.73m^2^) | 95 | |  |
| Total cholesterol (mg/dL) | 200 | |  |
| HDL cholesterol (mg/dL) | 60 | |  |
| Smoking status | No | |  |
| Diabetes | Yes | |  |
| Hypertension medication | No | |  |
| Statins | No | |  |
|  | PREVENT 10-year CVD risk=0.034 | | Recalibrated PREVENT CVD risk = 0.025 |
| **Step 2: Calculate relative risk (RR) for Unemployment status predictor (follow-up time analytically censored at 10 years)** | | | |
| 1. Cause-specific model for predictor + offset(log odds of recalibrated PREVENT risk) | $model 1=riskRegression∷CSC(Hist\left( surv\_yr ,status \right)\sim Employment Status+offset(\log odds(PREVENT risk recalibrated)))$ | | |
| 1. Predict 10-year risk based on predictor + offset(log odds of recalibrated PREVENT risk) | $model 2=riskRegression∷predictRisk(model1, times=10, cause=CVD)$ | | |
| 1. Ratio of mean predicted risk among those with the predictor vs those without | $Relative Risk \left( \mathrm{RR} \right)=\frac{\mathrm{Mean}\left( model 2 predicted risks, Predictor present \right)}{Mean(model 2 predicted risks, Predictor absent)}$ | | |
|  | $RR=\frac{\mathrm{Mean}\left( model 2 predicted risks, Predictor present \right)}{Mean(model 2 predicted risks, Predictor absent)}=\frac{5.022750\%}{1.076443\%}=\mathbf{4.66606}$ | | |
| **Step 3: Calculate population RR, as presented in Hageman et al^1^** | | | |
| 𝑝𝑜𝑝𝑢𝑙𝑎𝑡𝑖𝑜𝑛 𝑅𝑅 = (𝑝𝑟𝑒𝑣𝑎𝑙𝑒𝑛𝑐𝑒 𝑜𝑓 𝑝𝑟𝑒𝑑𝑖𝑐𝑡𝑜𝑟) ∗ 𝑅𝑅 𝑜𝑓 𝑝𝑟𝑒𝑑𝑖𝑐𝑡𝑜𝑟 + (1 − 𝑝𝑟𝑒𝑣𝑎𝑙𝑒𝑛𝑐𝑒) | | | |
| Unemployment | RR | Prevalence of Unemployment | Population RR |
|  | 4.66606 | 0.39856 | 2.46115 |
| **Step 4: Calculate updated predicted risk, as presented in Hageman et al^1^** | | | |
| For categorical variables: | | | |
| If predictor present: | $1-({1-recalibrated individual predicted risk)}^{\left( \frac{\mathrm{RR}}{population RR} \right)}$ | | |
| If predictor absent: | $1-({1-recalibrated individual predicted risk)}^{\left( \frac{1}{population RR} \right)}$ | | |
| Updated risk for Unemployed (predictor “present”): | $1-({1-0.025)}^{\left( \frac{4.66606}{2.36115} \right)}=0.049829 = 4.98\%$ | | |
| Updated risk for employed (predictor “absent”) | $1-({1-0.025)}^{\left( \frac{1}{2.36115} \right)}=0.010665 = 1. 07\%$ | | |

# Table 1 Demographic Characteristics for Sensitivity Analysis Samples

| **Supplementary Table 2. Demographic characteristics, PREVENT/PCE risk score**  **variables, and social determinants of health variables in sensitivity analysis A sample: complete-case CVD-free HANDLS population, excluding individuals < 40 years (n=1576)** | |
| --- | --- |
| **Variable** |  |
| 10-year cumulative incidence of CVD death  (95% CI) | 3.43% (2.61%, 4.41%) |
| Age, years | 52 (7) |
| Black | 886 (56%) |
| White | 690 (44%) |
| Female | 893 (57%) |
| **PREVENT and PCE risk score variables** | |
| BMI, kg/m^2^ | 30 (8) |
| Total cholesterol, mg/dL | 191 (42) |
| HDL cholesterol, mg/dL | 53 (17) |
| Systolic blood pressure, mmHg | 122 (18) |
| eGFR, mL/min/1.73m^2^ | 88 (18) |
| Current cigarette smoker | 761 (48%) |
| Diabetes | 282 (18%) |
| Use of antihypertensive medication | 536 (34%) |
| Use of lipid-lowering medication | 219 (14%) |
| **Social Determinants of Health** | |
| *Poverty Status* | |
| Below 125% of the 2004 Federal Poverty Guidelines level | 616 (39%) |
| *Education* |  |
| Less than high school education | 476 (31%) |
| Missing | 28 |
| *Homeownership* | |
| Owns own home | 704 (46%) |
| Rents home | 608 (40%) |
| Home owned or rented by friend or relative | 219 (14%) |
| Other | 5 (0.3%) |
| Missing | 40 |
| *Employment – dichotomous* | |
| Unemployed within the last month | 650 (42%) |
| Employed within the last month | 886 (58%) |
| Missing | 40 |
| *Three levels of unemployment* | |
| Voluntary unemployment/other | 170 (26%) |
| Involuntary unemployment, can’t find job | 135 (21%) |
| Involuntary unemployment, disabled/health-related reasons | 345 (53%) |
| Continuous variables are reported as mean (standard deviation)  Categorical variables are reported as n (%)  For variables with missing data, percentages are reported as a percent of the complete-case sample.  Voluntary unemployment/other includes individuals who reported the following reasons for unemployment: Taking care of house/kids or other caretaking; Doesn’t need or want to work; Student; Retired; Other | |

| **Supplementary Table 3. Demographic characteristics, PREVENT/PCE risk score variables, and social determinants of health variables in sensitivity analysis B sample (n=2094)** | |
| --- | --- |
| **Variable** |  |
| 10-year cumulative incidence of CVD death (95% CI) | 2.72% (2.09%, 3.49%) |
| Age, years | 48 (9) |
| Black | 1,179 (56%) |
| White | 915 (44%) |
| Female | 1,171 (56%) |
| **PREVENT and PCE risk score variables** |  |
| BMI, kg/m^2^ | 30 (8) |
| Total cholesterol, mg/dL | 188 (42) |
| HDL cholesterol, mg/dL | 53 (17) |
| Systolic blood pressure, mmHg | 120 (18) |
| eGFR, mL/min/1.73m^2^ | 91 (18) |
| Current cigarette smoker | 1,002 (48%) |
| Diabetes | 319 (15%) |
| Use of antihypertensive medication | 582 (28%) |
| Use of lipid-lowering medication | 227 (11%) |
| **Social Determinants of Health** |  |
| *Poverty Status* |  |
| Below 125% of the 2004 Federal Poverty Guidelines level | 823 (39%) |
| *Education* |  |
| Less than high school education | 639 (31%) |
| Missing | 38 |
| *Homeownership* |  |
| Owns own home | 863 (42%) |
| Rents home | 868 (43%) |
| Home owned or rented by friend or relative | 302 (15%) |
| Other | 6 (0.3%) |
| Missing | 55 |
| *Employment – dichotomous* |  |
| Unemployed within the last month | 810 (40%) |
| Employed within the last month | 1229 (60%) |
| Missing | 55 |
| *Three levels of unemployment* |  |
| Voluntary unemployment/other | 233 (28%) |
| Involuntary unemployment, can’t find job | 186 (23%) |
| Involuntary unemployment, disabled/health-related reasons | 391 (48%) |
| Continuous variables are reported as mean (standard deviation)  Categorical variables are reported as n (%)  For variables with missing data, percentages are reported as a percent of the complete-case sample  Voluntary unemployment/other includes individuals who reported the following reasons for unemployment: Taking care of house/kids or other caretaking; Doesn’t need or want to work; Student; Retired; Other | |

| **Supplementary Table 4. Demographic characteristics, PREVENT/PCE risk score variables, and social determinants of health variables in Quest-restricted sensitivity analysis C sample (n=1900)** | |
| --- | --- |
| **Variable** |  |
| 10-year cumulative incidence of CVD death (95% CI) | 2.68% (2.03%, 3.48% |
| Age, years | 48 (9) |
| Black | 1,039 (55%) |
| White | 861 (45%) |
| Female | 1,084 (57%) |
| **PREVENT and PCE risk score variables** |  |
| BMI, kg/m^2^ | 30 (8) |
| Total cholesterol, mg/dL | 188 (43) |
| HDL cholesterol, mg/dL | 53 (17) |
| Systolic blood pressure, mmHg | 120 (18) |
| eGFR, mL/min/1.73m^2^ | 91 (18) |
| Current cigarette smoker | 883 (46%) |
| Diabetes | 300 (16%) |
| Use of antihypertensive medication | 550 (29%) |
| Use of lipid-lowering medication | 219 (12%) |
| **Social Determinants of Health** |  |
| *Poverty Status* |  |
| Below 125% of the 2004 Federal Poverty Guidelines level | 721 (38%) |
| *Education* |  |
| Less than high school education | 568 (30%) |
| Missing | 37 |
| *Homeownership* |  |
| Owns own home | 816 (44%) |
| Rents home | 744 (40%) |
| Home owned or rented by friend or relative | 284 (15%) |
| Other | 5 (0.3%) |
| Missing | 51 |
| *Employment – dichotomous* |  |
| Unemployed within the last month | 719 (39%) |
| Employed within the last month | 1130 (61%) |
| Missing | 51 |
| *Three levels of unemployment* |  |
| Voluntary unemployment/other | 209 (29%) |
| Involuntary unemployment, can’t find job | 168 (23%) |
| Involuntary unemployment, disabled/health-related reasons | 342 (48%) |
| Continuous variables are reported as mean (standard deviation)  Categorical variables are reported as n (%)  For variables with missing data, percentages are reported as a percent of the complete-case sample  Voluntary unemployment/other includes individuals who reported the following reasons for unemployment: Taking care of house/kids or other caretaking; Doesn’t need or want to work; Student; Retired; Other | |

| **Supplementary Table 5. Demographic characteristics, PREVENT/PCE risk score variables, and social determinants of health variables in Quest-restricted sensitivity analysis D sample (n=1992)** | |
| --- | --- |
| **Variable** |  |
| 10-year cumulative incidence of CVD death (95% CI) | 2.66% (2.02%, 3.44%) |
| Age, years | 48 (9) |
| Black | 1,093 (55%) |
| White | 899 (45%) |
| Female | 1,128 (57%) |
| **PREVENT and PCE risk score variables** |  |
| BMI, kg/m^2^ | 30 (8) |
| Total cholesterol, mg/dL | 188 (43) |
| HDL cholesterol, mg/dL | 53 (17) |
| Systolic blood pressure, mmHg | 120 (18) |
| eGFR, mL/min/1.73m^2^ | 91 (18) |
| Current cigarette smoker | 932 (47%) |
| Diabetes | 308 (15%) |
| Use of antihypertensive medication | 554 (28%) |
| Use of lipid-lowering medication | 219 (11%) |
| **Social Determinants of Health** |  |
| *Poverty Status* |  |
| Below 125% of the 2004 Federal Poverty Guidelines level | 754 (38%) |
| *Education* |  |
| Less than high school education | 598 (31%) |
| Missing | 55 |
| *Homeownership* |  |
| Owns own home | 849 (44%) |
| Rents home | 787 (41%) |
| Home owned or rented by friend or relative | 296 (15%) |
| Other | 5 (0.3%) |
| Missing | 55 |
| *Employment – dichotomous* |  |
| Unemployed within the last month | 751(39%) |
| Employed within the last month | 1186 (61%) |
| Missing | 55 |
| *Three levels of unemployment* |  |
| Voluntary unemployment/other | 225 (30%) |
| Involuntary unemployment, can’t find job | 172 (23%) |
| Involuntary unemployment, disabled/health-related reasons | 354 (47%) |
| Continuous variables are reported as mean (standard deviation)  Categorical variables are reported as n (%)  For variables with missing data, percentages are reported as a percent of the complete-case sample  Voluntary unemployment/other includes individuals who reported the following reasons for unemployment: Taking care of house/kids or other caretaking; Doesn’t need or want to work; Student; Retired; Other | |

| **Supplementary Table 6. Discrimination of PCE and PREVENT in HANDLS, excluding individuals < 40 years, sensitivity analysis A sample** | | | | | |
| --- | --- | --- | --- | --- | --- |
| **Total population** | | | | | |
|  | **PCE** | | **PREVENT** | |  |
| N (CVD deaths by 10 years) | AUCt=10 (95% CI) | | | | p-value |
| 1576 (54) | 0.67 (0.59, 0.75) | | 0.73 (0.66, 0.80) | | 0.03 |
|  | Continuous NRI PREVENT vs PCE | | | |  |
|  | NRI (95% CI) | NRI event (95% CI) | | NRI non-event (95% CI) |  |
|  | 0.21 (-0.04,  0.48) | -0.11 (-0.36, 0.15) | | 0.32 (0.27, 0.37) |  |
| **Black persons** | | | | | |
| N (CVD deaths by 10 years) | AUCt=10 (95% CI) | | | | p-value |
| 886 (36) | 0.67 (0.58, 0.77) | | 0.74 (0.66, 0.82) | | 0.04 |
| **White persons** | | | | | |
| N (CVD deaths by 10 years) | AUCt=10 (95% CI) | | | | p-value |
| 690 (18) | 0.66 (0.52, 0.80) | | 0.70 (0.57, 0.82) | | 0.29 |
| The reported AUCt=10 is the AUC_2 value reported in the timeROC() R package. | | | | | |

# Discrimination and risk reclassification comparing PREVENT and PCE in Sensitivity Analysis Samples

| **Supplementary Table 7. Discrimination of PCE and PREVENT in HANDLS, sensitivity analysis B sample** | | | | | |
| --- | --- | --- | --- | --- | --- |
| **Total population** | | | | | |
|  | **PCE** | | **PREVENT** | |  |
| N (CVD deaths by 10 years) | AUC_t=10_ (95% CI) | | | | p-value |
| 2094 (57) | 0.72 (0.64, 0.79) | | 0.77 (0.72, 0.83) | | 0.01 |
|  | Continuous NRI PREVENT vs PCE | | | |  |
|  | NRI (95% CI) | NRI event (95% CI) | | NRI non-event (95% CI) |  |
|  | 0.38 (0.11, 0.64) | 0.09 (-0.19, 0.36) | | 0.29 (0.25, 0.33) |  |
| **Black persons** | | | | | |
| N (CVD deaths by 10 years) | AUC_t=10_ (95% CI) | | | | p-value |
| 1179 (37) | 0.71 (0.62, 0.80) | | 0.78 (0.70, 0.85) | | 0.02 |
| **White persons** | | | | | |
| N (CVD deaths by 10 years) | AUC_t=10_ (95% CI) | | | | p-value |
| 915 (20) | 0.72 (0.61, 0.84) | | 0.77 (0.68, 0.86) | | 0.13 |
| The reported AUC_t=10_ is the AUC_2 value reported in the timeROC() R package. | | | | | |

| **Supplementary Table 8. Discrimination of PCE and PREVENT in HANDLS, Quest-restricted sensitivity analysis C sample** | | | | | |
| --- | --- | --- | --- | --- | --- |
| **Total population** | | | | | |
|  | **PCE** | | **PREVENT** | |  |
| N (CVD deaths by 10 years) | AUC_t=10_ (95% CI) | | | | p-value |
| 1900 (51) | 0.72 (0.64, 0.79) | | 0.77 (0.70, 0.83) | | 0.04 |
|  | Continuous NRI PREVENT vs PCE | | | |  |
|  | NRI (95% CI) | NRI event (95% CI) | | NRI non-event (95% CI) |  |
|  | 0.18 (-0.10, 0.47) | -0.02 (-0.30, 0.26) | | 0.20 (0.16, 0.25) |  |
| **Black persons** | | | | | |
| N (CVD deaths by 10 years) | AUC_t=10_ (95% CI) | | | | p-value |
| 1039 (33) | 0.72 (0.62, 0.81) | | 0.77 (0.69, 0.85) | | 0.08 |
| **White persons** | | | | | |
| N (CVD deaths by 10 years) | AUC_t=10_ (95% CI) | | | | p-value |
| 861 (18) | 0.71 (0.59, 0.83) | | 0.76 (0.66, 0.86) | | 0.14 |
|  |  | |  | |  |
| The reported AUC_t=10_ is the AUC_2 value reported in the timeROC() R package. | | | | | |

| **Supplementary Table 9. Discrimination of PCE and PREVENT in HANDLS, Quest-restricted sensitivity analysis D sample** | | | | | |
| --- | --- | --- | --- | --- | --- |
| **Total population** | | | | | |
|  | **PCE** | | **PREVENT** | |  |
| N (CVD deaths by 10 years) | AUC_t=10_ (95% CI) | | | | p-value |
| 1992 (53) | 0.72 (0.65, 0.80) | | 0.77 (0.72, 0.83) | | 0.02 |
|  | Continuous NRI PREVENT vs PCE | | | |  |
|  | NRI (95% CI) | NRI event (95% CI) | | NRI non-event (95% CI) |  |
|  | 0.18 (-0.10, 0.46) | -0.02 (-0.29, 0.26) | | 0.20 (0.16, 0.24) |  |
| **Black persons** | | | | | |
| N (CVD deaths by 10 years) | AUC_t=10_ (95% CI) | | | | p-value |
| 1093 (33) | 0.72 (0.62, 0.81) | | 0.78 (0.70, 0.85) | | 0.07 |
| **White persons** | | | | | |
| N (CVD deaths by 10 years) | AUC_t=10_ (95% CI) | | | | p-value |
| 899 (20) | 0.72 (0.61, 0.84) | | 0.77 (0.68, 0.86) | | 0.12 |
|  |  | |  | |  |
| The reported AUC_t=10_ is the AUC_2 value reported in the timeROC() R package. | | | | | |

# Forest plots for SDoH associations with CVD mortality in Sensitivity Analysis Samples

**Figure S5. Hazard ratios (HR) (95% confidence intervals) for associations between select individual-level social determinants of health variables and CVD mortality in HANDLS sensitivity analysis A sample.** Model 1: adjusted for age and sex as reported at the baseline visit. Model 2: adjusted for the log-odds of the recalibrated PREVENT risk estimate. HR (95% CI) are plotted on the log-scale.

Although the Schoenfeld residual plots for each of the Model 2 analyses appeared generally visually consistent with the proportional hazards assumption, the p-value for the covariate “log-odds of PREVENT” was <0.05, so we also performed Model 2 analyses accounting for time-dependent effects of log-odds of PREVENT, which resulted in very similar results to the original analyses:

HR (95% CI):

Less than high school vs high school or higher = 1.33 (0.77, 2.33)

Below vs above federal poverty delimiter = 1.50 (0.87, 2.57)

Rent or other vs own home = 1.47 (0.84, 2.58)

Unemployed vs employed = 3.22 (1.70, 6.10)

**Figure S6. Hazard ratios (HR) (95% confidence intervals) for associations between select individual-level social determinants of health variables and CVD mortality in HANDLS sensitivity analysis B sample.** Model 1: adjusted for age and sex as reported at the baseline visit. Model 2: adjusted for the log-odds of the recalibrated PREVENT risk estimate. HR (95% CI) are plotted on the log-scale.

**Figure S7. Hazard ratios (HR) (95% confidence intervals) for associations between select individual-level social determinants of health variables and CVD mortality in HANDLS sensitivity analysis C sample.** Model 1: adjusted for age and sex as reported at the baseline visit. Model 2: adjusted for the log-odds of the recalibrated PREVENT risk estimate. HR (95% CI) are plotted on the log-scale.

**Figure S8. Hazard ratios (HR) (95% confidence intervals) for associations between select individual-level social determinants of health variables and CVD mortality in HANDLS sensitivity analysis D sample.** Model 1: adjusted for age and sex as reported at the baseline visit. Model 2: adjusted for the log-odds of the recalibrated PREVENT risk estimate. HR (95% CI) are plotted on the log-scale.

# Discrimination and risk reclassification for PREVENT+Unemployment updating versus PREVENT in Sensitivity Analysis Samples

| **Supplementary Table 10. Discrimination of PREVENT and updated PREVENT + Unemployment in HANDLS, sensitivity analysis A sample, n=1536 with complete employment data** | | | | | |
| --- | --- | --- | --- | --- | --- |
| ***Total population*** | | | | | |
|  | **PREVENT** | | **PREVENT+Unemployment** | |  |
| *N (CVD deaths by 10 years)* | *AUC_t=10_ (95% CI)* | | | | *p-value* |
| 1536 (52) | 0.72 (0.65, 0.80) | | 0.76 (0.69, 0.82) | | 0.10 |
|  | *Continuous NRI PREVENT vs PCE* | | | |  |
|  | *NRI (95% CI)* | *NRI event (95% CI)* | | *NRI non-event (95% CI)* |  |
|  | 0.68 (0.43, 0.91) | 0.50 (0.25, 0.74) | | 0.18 (0.13, 0.23) |  |
| ***Black persons*** | | | | | |
| *N (CVD deaths by 10 years)* | *AUC_t=10_ (95% CI)* | | | | *p-value* |
| 883 (36) | 0.74 (0.66, 0.83) | | 0.77 (0.69, 0.84) | | 0.31 |
| ***White persons*** | | | | | |
| *N (CVD deaths by 10 years)* | *AUC_t=10_ (95% CI)* | | | | *p-value* |
| 653 (16) | 0.68 (0.55, 0.82) | | 0.73 (0.61, 0.86) | | 0.21 |
| ***Unemployed persons*** | | | | | |
| *N (CVD deaths by 10 years)* | *AUC_t=10_ (95% CI)* | | | | *p-value* |
| 650 (39) | 0.70 (0.61, 0.79) | | | | -- |
| ***Employed persons*** | | | | | |
| *N (CVD deaths by 10 years)* | *AUC_t=10_ (95% CI)* | | | | *p-value* |
| 886 (13) | 0.68 (0.55, 0.81) | | | | -- |
| The reported AUC_t=10_ is the AUC_2 value reported in the timeROC() R package. | | | | | |

| **Supplementary Table 11. Discrimination of PREVENT and updated PREVENT + Unemployment in HANDLS, sensitivity analysis B sample, n=2039 with complete employment data** | | | | | |
| --- | --- | --- | --- | --- | --- |
| **Total population** | | | | | |
|  | **PREVENT** | | **PREVENT+Unemployment** | |  |
| N (CVD deaths by 10 years) | AUC_t=10_ (95% CI) | | | | p-value |
| 2039 (55) | 0.77 (0.71, 0.83) | | 0.79 (0.74, 0.85) | | 0.23 |
|  | Continuous NRI PREVENT vs PCE | | | |  |
|  | NRI (95% CI) | NRI event (95% CI) | | NRI non-event (95% CI) |  |
|  | 0.68 (0.43, 0.91) | 0.45 (0.21, 0.67) | | 0.22 (0.18, 0.27) |  |
| **Black persons** | | | | | |
| N (CVD deaths by 10 years) | AUC_t=10_ (95% CI) | | | | p-value |
| 1174 (37) | 0.78 (0.70, 0.85) | | 0.80 (0.73, 0.87) | | 0.28 |
| **White persons** | | | | | |
| N (CVD deaths by 10 years) | AUC_t=10_ (95% CI) | | | | p-value |
| 865 (18) | 0.76 (0.66, 0.86) | | 0.78 (0.69, 0.87) | | 0.61 |
| **Unemployed persons** | | | | | |
| N (CVD deaths by 10 years) | AUC_t=10_ (95% CI) | | | | p-value |
| 810 (40) | 0.73 (0.65, 0.81) | | | | -- |
| **Employed persons** | | | | | |
| N (CVD deaths by 10 years) | AUC_t=10_ (95% CI) | | | | p-value |
| 1229 (15) | 0.77 (0.68, 0.87) | | | | -- |
| The population RR used for PREVENT+Unemployment model updating was 2.21.  The reported AUC_t=10_ is the AUC_2 value reported in the timeROC() R package. | | | | | |

| **Supplementary Table 12. Discrimination of PREVENT and updated PREVENT + Unemployment in HANDLS, Quest-restricted sensitivity analysis C sample, n=1849 with complete employment data** | | | | | |
| --- | --- | --- | --- | --- | --- |
| **Total population** | | | | | |
|  | **PREVENT** | | **PREVENT+Unemployment** | |  |
| N (CVD deaths by 10 years) | AUC_t=10_ (95% CI) | | | | p-value |
| 1849 (49) | 0.76 (0.70, 0.83) | | 0.79 (0.73, 0.85) | | 0.16 |
|  | Continuous NRI PREVENT vs PCE | | | |  |
|  | NRI (95% CI) | NRI event (95% CI) | | NRI non-event (95% CI) |  |
|  | 0.71 (0.43, 0.97) | 0.47 (0.20, 0.72) | | 0.24 (0.20, 0.28) |  |
| **Black persons** | | | | | |
| N (CVD deaths by 10 years) | AUC_t=10_ (95% CI) | | | | p-value |
| 1034 (33) | 0.77 (0.69, 0.85) | | 0.79 (0.72, 0.86) | | 0.38 |
| **White persons** | | | | | |
| N (CVD deaths by 10 years) | AUC_t=10_ (95% CI) | | | | p-value |
| 815 (16) | 0.75 (0.64, 0.86) | | 0.79 (0.69, 0.89) | | 0.26 |
| **Unemployed persons** | | | | | |
| N (CVD deaths by 10 years) | AUC_t=10_ (95% CI) | | | | p-value |
| 719 (36) | 0.73 (0.64, 0.81) | | | | -- |
| **Employed persons** | | | | | |
| N (CVD deaths by 10 years) | AUC_t=10_ (95% CI) | | | | p-value |
| 1130 (13) | 0.75 (0.64, 0.86) | | | | -- |
| The population RR used for PREVENT+Unemployment model updating was 2.31  The reported AUC_t=10_ is the AUC_2 value reported in the timeROC() R package. | | | | | |

| **Supplementary Table 13. Discrimination of PREVENT and updated PREVENT + Unemployment in HANDLS, Quest-restricted sample sensitivity analysis D sample, n=1937 with complete employment data** | | | | | |
| --- | --- | --- | --- | --- | --- |
| **Total population** | | | | | |
|  | **PREVENT** | | **PREVENT+Unemployment** | |  |
| N (CVD deaths by 10 years) | AUC_t=10_ (95% CI) | | | | p-value |
|  | 0.77 (0.71, 0.83) | | 0.79 (0.73, 0.85) | | 0.27 |
| 1937 (51) | Continuous NRI PREVENT vs PCE | | | |  |
|  | NRI (95% CI) | NRI event (95% CI) | | NRI non-event (95% CI) |  |
|  | 0.65 (0.37, 0.91) | 0.41 (0.14, 0.66) | | 0.24 (0.20, 0.29) |  |
| **Black persons** | | | | | |
| N (CVD deaths by 10 years) | AUC_t=10_ (95% CI) | | | | p-value |
| 1088 (33) | 0.78 (0.70, 0.85) | | 0.79 (0.72, 0.87) | | 0.35 |
| **White persons** | | | | | |
| N (CVD deaths by 10 years) | AUC_t=10_ (95% CI) | | | | p-value |
| 849 (18) | 0.76 (0.66, 0.86) | | 0.78 (0.69, 0.87) | | 0.58 |
| **Unemployed persons** | | | | | |
| N (CVD deaths by 10 years) | AUC_t=10_ (95% CI) | | | | p-value |
| 751 (36) | 0.73 (0.65, 0.81) | | | | -- |
| **Employed persons** | | | | | |
| N (CVD deaths by 10 years) | AUC_t=10_ (95% CI) | | | | p-value |
| 1186 (15) | 0.77 (0.68, 0.87) | | | | -- |
| The population RR used in PREVENT+Unemployment model updating was 2.09.  The reported AUC_t=10_ is the AUC_2 value reported in the timeROC() R package. | | | | | |

References

1. Hageman SHJ, Petitjaen C, Pennells L, et al. Improving 10-year cardiovascular risk prediction in apparently healthy people: flexible addition of risk modifiers on top of SCORE2. Eur J Prev Cardiol. Oct 26 2023;30(15):1705-1714. doi:10.1093/eurjpc/zwad187. PMC10600319
